# Efficacy of the erector spinae plane (ESP) block for quality of recovery in posterior thoraco-lumbar spinal decompression surgery: study protocol for a randomised controlled trial

**DOI:** 10.1186/s13063-021-05101-2

**Published:** 2021-02-17

**Authors:** Dylan T. Finnerty, Donal J. Buggy

**Affiliations:** 1 Division of Anaesthesiology, Mater Misericordiae University Hospital, Eccles Street, Dublin, D07 R2WY Ireland; 2 School of Medicine, University College Dublin, Dublin, Ireland; 3 EU COST Action 15204 Euro-Periscope, Brussels, Belgium; 4 Outcomes Research, Cleveland Clinic, Cleveland, OH USA

**Keywords:** Erector spinae plane block, Spine surgery, Quality of recovery, Morbidity, Analgesia

## Abstract

**Background:**

Spinal surgery can be associated with significant postoperative pain. Erector spinae plane (ESP) block is a new regional anaesthesia technique, which promises effective postoperative analgesia compared with systemically administered opioids, but has never been evaluated in terms of patient-centred outcomes such as quality of recovery and overall morbidity after major thoraco-lumbar spinal surgery.

**Methods:**

We are conducting a prospective, randomised, double-blind trial in two hospitals in the Republic of Ireland. The sample size will be 50 patients (25 in the intervention group and 25 in the control group). Randomisation will be done using computer-generated concealed envelopes. Both patients and investigators collecting outcome data will be masked to group allocation. Participants will be male or female, aged 18 years and over, capable of providing informed consent and ASA grade I–IV. Patients scheduled to undergo posterior approach thoraco-lumbar decompression surgery involving 2 or more levels will be recruited to the study. Participants randomised to the intervention arm of the study will receive bilateral ultrasound-guided ESP block totalling 40 ml 0.25% levo-bupivcaine (20 ml each side), post induction of general anaesthesia and before surgical incision. The control group will not receive an ESP block. Both groups will receive the same standardised analgesic protocol both intra- and postoperatively. The primary outcome will be the quality of recovery at 24 h postoperatively as determined by the QoR-15 score. This score is determined by a questionnaire which measures patient responses to 15 subjective parameters, each response graded on a scale from 0 to 10. The maximum score achievable is 150 with a potential minimum score of 0. Higher scores indicate a higher quality of recovery experience.

Secondary outcomes will include area under the curve (AUC) of VRS pain versus time at rest and on movement up to 24 h postoperatively, 24 h opioid consumption, time to first analgesia in recovery, length of stay (LOS), incidence and severity of postoperative complications as measured by the Comprehensive Complication Index (CCI) score.

**Discussion:**

To the best of our knowledge, this will be the first randomised control trial to examine the efficacy and safety of the ESP block in terms of patient-centred outcomes in the setting of major spinal surgery. The QoR-15 is a validated means of assessing the quality of recovery after surgery and gives a more holistic assessment of the recovery experience from the patient’s point of view.

**Trial registration:**

This trial is pre-registered on ClinicalTrials.gov reference number NCT04370951. Registered on 30 April 2020. All items from the World Health Organisation Trial Registration Data Set have been included.

**Supplementary Information:**

The online version contains supplementary material available at 10.1186/s13063-021-05101-2.

## Background

Major spine surgery is acknowledged to be associated with moderate to severe postoperative pain. A prospective cohort study of patients undergoing spinal surgery reported median verbal response scale (VRS) pain scores ranging from 5 to 7 on the first postoperative day [1]. Severe postoperative pain is associated with considerable morbidity, prolonged length of in-hospital stay (LOS), increased opiate requirements and prolonged time to mobilisation.

Recently, a novel regional anaesthesia technique has been described for patients undergoing spinal surgery. First described by Forero in 2016 for the treatment of thoracic neuropathic pain, the erector spinae plane (ESP) block involves depositing local anaesthesia under ultrasound guidance on the transverse process of the thoracic vertebrae, deep to the erector spinae muscle complex [2]. Cadaveric and MRI studies suggest that ESP can block the posterior rami of the spinal nerves in addition to the anterior spinal nerve rami to the paravertebral and epidural spaces, although this seems less consistent [3–5]. The posterior ramus of the spinal nerves provides sensory innervation of the paraspinal muscles, soft tissue and skin at the level at which it emerges [6]. This is of particular relevance in spinal surgery, because these structures must be incised and retracted in order to gain appropriate surgical exposure.

The evidence base for ESP in spine surgery is sparse. The published literature that currently exists reports lower VRS pain scores postoperatively among those patients receiving ESP compared with systemic opioids [7–10]. While lower pain scores are important, they may not be perceived by the patient as a better recovery if they are accompanied by other debilitating side effects such as subjective lack of wellness, depression, nausea, constipation or delirium. Pain scores alone are an insensitive marker of the quality of a patient’s recovery after major surgery.

Recent best practice in anaesthesia and perioperative medicine research has been to adopt a more holistic and patient-centred approach to the evaluation of the recovery experience. The QoR-15 score is internationally recognised as a validated means of assessing patients’ quality of recovery after surgery [11]. The score assesses 5 domains of patient-reported health status: pain, physical comfort, physical independence, psychological state and emotional state to give a holistic assessment of the patient’s overall recovery experience.

Currently, opiate therapy is the primary treatment option for patients suffering acute postoperative pain after major spinal surgery. Adverse events associated with high opioid requirements postoperatively are well documented and include nausea, constipation, respiratory depression, lower respiratory tract infections and drug dependency [12]. Available evidence to date suggests the ESP block may have opioid-sparing properties [7, 9, 10], making it an attractive option in spine surgery that merits further investigation.

Additionally, the ESP block appears to be relatively simple to perform and is associated with few adverse events [13]. Alternative regional techniques such as epidural and paravertebral anaesthesia are technically more challenging to perform and are associated with significant safety risks, such as epidural haematoma or pneumothorax [14–16]. While the ESP block has been adopted enthusiastically by many anaesthesiologists, evidence showing its efficacy in improving the recovery experience and reducing morbidity for patients is lacking and therefore this clinical trial is warranted.

We hypothesise that ESP block provides superior quality of recovery and reduced morbidity compared with systemic analgesia alone. This is based on the scientific rationale that it blocks both posterior and anterior rami of the spinal nerves, thereby providing extensive block and better analgesia.

We propose a prospective, two-centre, randomised control trial of 50 patients scheduled for posterior approach thoraco-lumbar spinal surgery. The co-primary outcomes will be QoR-15 score at 24 h postoperatively and postoperative complications as measured by the Comprehensive Complication Index (CCI) score.

Secondary outcomes include area under the curve (AUC) of VRS pain versus time at rest and on movement up to 24 h postoperatively, 24 h opioid consumption, time to first analgesia in recovery and length of stay (LOS). This paper describes the protocol for the intended trial.

## Methods and analysis

**Fig. 1:**
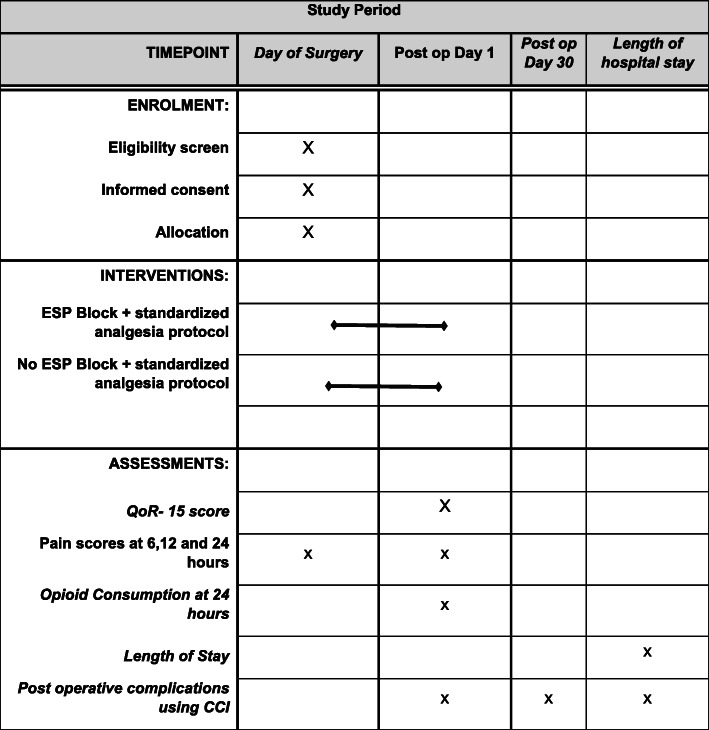
Overview of assessment. Recommendations for Interventional Trials (SPIRIT) Figure showing the schedule of enrolment, interventions and assessment

The trial protocol has been prepared and is reported in accordance with the SPIRIT PRO extension reporting guidelines [17]. The schedule of enrolments, interventions and assessments is outlined in Fig. 1. The SPIRIT checklist is included as an additional file.

### Study objectives

#### Primary aims

We aim to complete a randomised control trial to test the hypothesis that the erector spinae plane block (combined with systemic analgesia) provides superior quality of recovery as measured by the QoR-15 score.

#### Secondary aims

Secondary aims are to discover whether the ESP block has favourable outcomes on other metrics of patient-centred patient outcome: area under the curve (AUC) of VRS pain versus time at rest and on movement up to 24 h postoperatively, 24 h opioid consumption, time to first analgesia in recovery, length of stay (LOS) and incidence and severity of postoperative complications as measured by the Comprehensive Complication Index (CCI) score.

### Study design

This is a prospective, double-blind (participant and investigator), randomised controlled clinical trial. Two centres will participate. Recruitment commenced on 1 May 2020, and total recruitment is expected to take 9 months.

### Study setting

The study setting comprises two tertiary level hospitals in the Republic of Ireland. Each hospital performs over 200 spinal surgeries per year.

### Randomisation and blinding

Patients will be randomised to one of the trial groups using computer-generated random number tables. Numbers ending in an even number integer will be designated to receive an ESP block; odd numbers will be allocated to the control group. The patient study number and group allocation will be typed on separate pages, folded and concealed in sequentially numbered sealed opaque envelopes. Block randomisation in groups of 6 will be applied to ensure an even number in each group as the study progresses. The groups will be named “ESP” erector spinae plane and “control”. A randomisation key will be held by an independent third party. Investigators will not have access to the randomisation key until all data is collected.

After induction of general anaesthesia, the envelope will be opened by the treating anaesthesiologist to reveal the group allocation. The investigators, patients and researchers involved in data collection will be masked to group allocation. The attending anaesthesiologist will not be blinded to the group allocation. Group allocation will be revealed immediately if deemed clinically necessary, for example, if a concern regarding local anaesthetic toxicity arises.

### Selection of participants

#### Recruitment

Potential participants will be screened by the surgical and anaesthetic teams who will then inform a member of the research team of the potential availability of the patient for the trial. A member of the research team will then approach the patient, confirm the initial screening for suitability and then obtain informed consent for study participation if the patient wishes to be involved in the study. Participants will be informed that their participation in the study is entirely voluntary and they are free to withdraw from the study at any time and this will have no bearing on the quality of care they receive. Patients who are unable to give informed consent due to cognitive impairment will be excluded from the trial. Patients who do not speak English will be enrolled to the trial if an interpreter is present and the patient gives informed consent through the interpreter.

Active participation in the study will be until 30 days postoperatively. At 24 h, patients will be asked to complete the QoR-15 questionnaire.

Postoperative complications will be graded using the Comprehensive Complication Index calculator (CCI^R^). This data will be acquired by using our electronic patient record system to remotely track the progress of our patients in the postoperative period. Patient progression will be monitored for the entirety of their hospital stay or for 30 days post surgery (whichever is longer).

Once the patient is discharged from hospital, there will be no further data collection. Where there is a breach in trial protocol this data will not be analysed.

### Inclusion criteria

- Male and female aged > 18
- Able to provide written informed consent
- ASA grade I–IV
- Planned posterior approach spinal surgery involving 2 or more levels in the lumbar or thoracic regions

#### Exclusion criteria

- Absence of or inability to give informed consent
- Pre-existing infection at block site
- Severe coagulopathy
- Allergy to local anaesthesia (or any other contraindication to block performance, e.g. concern re local anaesthesia toxicity)
- Previous history of opiate abuse
- Pre-existing chronic pain condition
- Pre-existing dementia (due to the need to co-operate in completing QoR-15 score day after surgery)
- Surgery involving cervical vertebrae
- Surgery due to queried or confirmed malignancy
- Surgery involving 5 or more vertebral levels
- Postoperative admission to ICU for continued ventilation

### Standard care

Standard care will be identical in both groups. The only difference will be that one group will receive an erector spinae plane block and the other will not. Airway management strategy will be at the discretion of the treating anaesthesiologist. However, as patients will be in the prone position, all patients will be intubated with a reinforced endotracheal tube. Ventilation strategy, choice of haemodynamic monitoring and venous access will also be at the discretion of the attending anaesthesiologist. The haemodynamic goal will be to maintain systolic blood pressure within 20% of the baseline. Persistent intraoperative elevations above this point will trigger oxycodone administration intravenously. The frequency and dosage of this will be at the discretion of the treating anaesthesiologist.

### Concomitant medication

Post surgery, patients will be transferred to the post anaesthesia care unit (PACU) and then to ward level, once PACU discharge criteria are met. Patients will be prescribed oxycodone 1–2 mg IV as required for postoperative pain in PACU until the verbal rating scale (VRS) pain score is < 2 in accordance with our hospital policy. On discharge to the ward, all patients will be prescribed paracetamol 1 g IV 6 hourly, ibuprofen 400 mg orally 8 hourly and oxycodone immediate release 5–10 mg 2 hourly as required for rescue analgesia unless contraindicated. Ondansetron 4 mg PO/IV 8 hourly as required will be prescribed for treatment of nausea or vomiting in the PACU or on discharge to the ward. Remifentanil and ketamine will not be used in any patient enrolled in the study.

### Concomitant treatment

Surgery will be performed by suitably qualified and experienced spinal surgeons. Surgical technique will vary as deemed necessary for each case. No other forms of analgesia (e.g. intrathecal opioids, epidural analgesia) will be used during the trial.

### Study intervention

Participants randomised to the ESP group will receive an ultrasound guided bilateral ESP block post induction of general anaesthesia and prior to commencement of surgery. Blocks will be performed under full asepsis with patients in the prone position. Twenty millilitres of 0.25% levobupivacaine will be injected bilaterally at the level corresponding to the mid-point of the range of surgical dermatomes likely to be affected by the surgery. Typically, ESP will be administered at T6 level (total 40 ml). The ESP block will be performed as follows:

The approximate midpoint of the intended surgical incision will be identified first. The ultrasound transducer will then be placed approximately to 2–3 cm lateral to the midline in a longitudinal orientation to identify the hyperechoic line of the transverse process with its associated acoustic shadow. After identification of trapezius, rhomboid major and erector spinae muscle groups superficial to the transverse process, an echogenic needle will be advanced in a cranio-caudal direction. The needle tip will be advanced until it is located in the interfascial plane deep to the erector spinae muscle group and superficial to the transverse process. Once in position, 20 ml 0.25% levobupivicaine will be injected under ultrasound guidance. Correct needle tip position will be confirmed by the presence of linear spread between the transverse process and the erector spinae muscle group. The same process will then be repeated on the opposite side at the same vertebral level. Because patients will be under general anaesthesia during the block performance, no formal dermatomal sensory testing of block efficacy will be performed. Surgery will commence once the block is completed. There will be no further intervention to the routine conduct of surgery and anaesthesia after this point. Blocks will be performed by anaesthesiologists with subspeciality training in regional anaesthesia. To improve adherence to the intervention protocol, anaesthesiologists will be asked to document the block performance in the anaesthesiology patient record. Although the ESP has been established as a safe means of providing analgesia in spine surgery, we will make all our data available to local data monitoring committee.

### Patient and process characteristics

The following data will be recorded in the perioperative period:

#### Preoperative

- Age
- BMI
- Pre induction blood pressure and heart rate
- Intended surgery

#### Intraoperative

- Timing/duration of surgery anaesthesia
- Haemodynamic parameters (highest and lowest BP and HR recorded)
- Total opioid given

#### Postoperative

- Surgical or anaesthesia-related complications (including complications related to ESP block)
- Pain scores at rest and on movement at 6, 12 and 24 h
- Analgesia use in the first 24 h
- Time to first analgesia
- Antiemetic usage
- QoR-15 score
- Length of stay

All patient data collected will be handled in accordance with European Union General Data Protection Regulations (EU 2016/679).

### Outcome measures

Quality of recovery will be measured using the QoR-15 questionnaire. Postoperative pain will be quantified using a verbal response scale (VRS) ranging from 0 to 10, where 0 equals “no pain at all” and 10 equals “the worst pain imaginable”. Reported pain scores will then be plotted against time to give the AUC of pain versus time. Pain scores will be recorded at 6, 12 and 24 h post surgery at rest and on movement.

Postoperative complications will be graded using the Comprehensive Complication Index calculator (CCI^R^). This data will be acquired using our electronic patient record system to remotely track the progress of our patients in the postoperative period. Patient progression will be monitored for the entirety of their hospital stay or for 30 days post surgery (whichever is longer).

### Statistical considerations

Data will be inspected and tested for distribution according to the Kolmogorov-Smirnov test. Normally distributed data will be compared between study arms using the unpaired *t* test, whereas non-normally distributed data will be compared using the Mann-Whitney *U* test. All data will be summarised as mean + SD or median (25–75% range) as appropriate.

### Sample size and justification

The primary outcome of this study will be the QoR-15 score at 24 h postoperatively. The established minimum clinically important difference in QoR-15 is 8.0 and the mean SD of QoR-15 scores after major spinal surgery is in the order of 14 [range of QoR score is 1–150]. Therefore, assuming type I error = 0.05 and type II error = 0.2 (80% power to detect this difference), then *n* = 25 patients will be required in each group. To accommodate for participants who may withdraw from the study, we will aim to recruit *n* = 26 patients to each study arm.

### Patient and public involvement

**Fig. 2:**
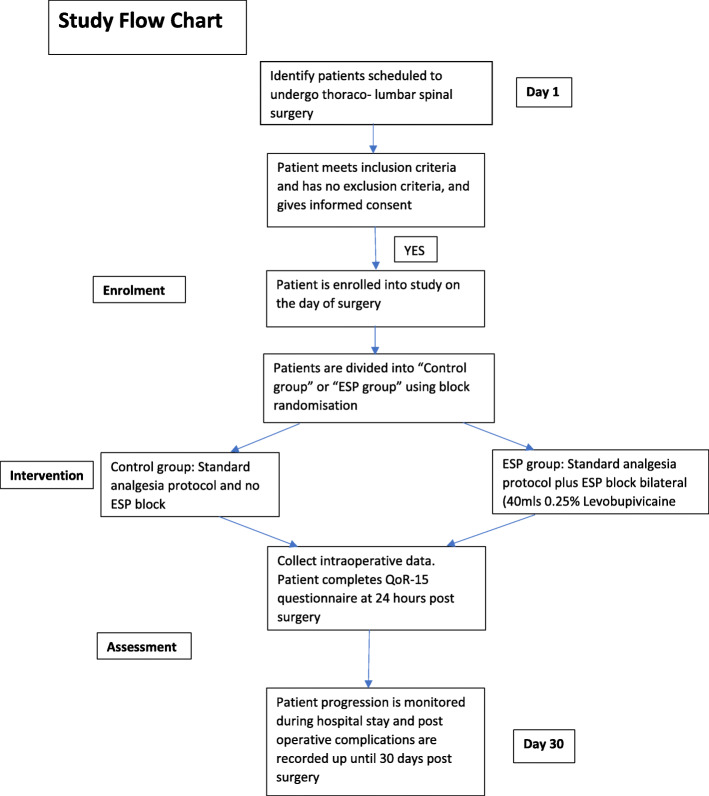
Study flow chart outlining time scale of enrolment, intervention and assessment

A patient information leaflet was written in “plain English” for trial participants. This was written in collaboration with and approved by our patient representative group. On the advice of the same group, we chose the QoR-15 questionnaire as opposed to the QoR-40 as the former was deemed to be easier for patients to read and comprehend. We also consulted with our patient advisory group on the drafting of our consent form. On their advice, we included a statement to say that their data protection rights would not be affected by participating in the study (Fig. 2).

## Discussion

We are conducting this multicentre, randomised control trial to investigate the efficacy of ESP in thoraco-lumbar spine surgery in terms of quality of recovery. The ESP block has become increasingly popular in recent times, and its use has been reported in a range of thoracic and abdominal surgery [18–20]. Its use in spinal surgery is less well documented and existing trials have used the traditional end points of opioid consumption and pain scores to assess its efficacy [7, 10, 21]. To the best of our knowledge, no trial to date has examined the impact of the ESP block on quality of recovery in spine patients.

Spine surgery is associated with significant postoperative pain and often a protracted recovery period. Current evidence suggests that the implementation of Enhanced Recovery After Surgery (ERAS) programmes in spine surgery may lead to improvements in functional recovery, length of stay, opioid use, complications and readmissions [22]. Because of its reputed analgesic benefits, ESP block may have a role as part of an ERAS programme and merits further investigation. By blocking the posterior rami of the spinal nerves, many structures traumatised during posterior approach spinal surgery could be targeted for alleviation by an ESP block.

More than 70% of patients presenting for major spinal surgery will already be taking opioids for pain relief. Preoperative opioid use combined with poorly controlled pain scores postoperatively have been identified as risk factors associated with opioid misuse during the postoperative period [23]. Given current public health concerns regarding addiction to prescription opioids administered postoperatively, particularly in the USA, there is a real need to focus our attention towards non-opioid based adjunctive analgesic strategies. Although ESP is a new regional anaesthetic technique, a recent meta-analysis provides moderate-quality evidence that the ESP block can reduce postoperative pain scores and opioid requirements after a range of surgeries [24]. The ESP block provides an exciting opportunity for investigators to possibly make a meaningful impact in patient-centred postoperative outcomes for spine surgery patients.

This protocol will result in the attending anaesthesiologist not being blinded to the group allocation as they will be required to perform the block. This is as an unavoidable situation, as for safety reasons will we feel that the treating anaesthesiologist should be aware that their patient has received 40 ml of 0.25% levobupivacaine. However, the primary outcome is the QoR-15 score at 24 h postoperatively which will be assessed by study members blinded to the group allocation.

We have chosen to exclude patients requiring surgical intervention of five or more vertebral levels. The rational for this decision comes from cadaveric studies, which suggest the maximum spread of local anaesthetic from a single injection ESP block to be approximately 5 levels [25]. Therefore, surgical dissection beyond this point is unlikely to benefit from a single shot technique, as proposed in this trial.

Pain scores and quality of recovery will be recorded up to 24 h postoperatively. We acknowledge that for many patients having major spinal surgery their recovery will continue beyond this point. We believe that any analgesic benefit derived from a single dose of local anaesthesia is unlikely to persist beyond 24 h, and so we will not assess pain scores after this point. However, length of stay and postoperative complications will continue to be monitored after this period.

ESP blocks will be done under general anaesthesia, therefore formal dermatomal assessment of block function will not be performed. This raises the possibility that some blocks may not be fully effective. However, the practice of administering these blocks under ultrasound guidance after induction of general anaesthesia is consistent with routine clinical practice, and therefore our findings should still be applicable to widespread clinical practice.

We do not intend to assess patients’ preoperative QoR-15 in this study. Consequently, we will not have a baseline from which to compare postoperative scores and this we accept is a limitation of the study. Nonetheless, QoR-15 was designed for postoperative use, and we will apply this tool to both randomised cohorts equally. Further, the ability of QoR-15 in the immediate preoperative period to give an accurate baseline has been questioned [26].

Recovery from spinal surgery can be an arduous journey for our patients. By employing a patient-centred focus to our trial design, we hope to evaluate whether this relatively straightforward regional anaesthetic intervention can facilitate recovery and improve safety for patients by reducing postoperative morbidity and complications. This trial is designed to answer that question.

### Trial status

As of May 11, 2020, 4 patients have been enrolled in this trial. Recruitment began on 1 May 2020 and is expected to be completed by 31 January 2021. This is protocol version 1 dated 3 April 2020.

## Supplementary Information

**Additional file 1.** Reporting checklist for protocol of a clinical trial.

## Data Availability

Data will be available upon reasonable request.

## Abbreviations

AUC: Area under the curve
CCI: Comprehensive Complication Index
ERAS: Enhanced Recovery After Surgery
ESP: Erector spinae plane
LOS: Length of in hospital stay
PACU: Post anaesthesia care unit
QoR-15: Quality of Recovery-15 item questionnaire
VRS: Verbal response scale
